# Incumbents’ Capabilities for Sustainability-Oriented Innovation in the Norwegian Food Sector—an Integrated Framework

**DOI:** 10.1007/s43615-022-00234-1

**Published:** 2022-11-25

**Authors:** Antje Gonera, Hilde Andrea Nykamp, Laura Carraresi

**Affiliations:** 1 Department of Innovation, Consumer and Sensory Science, Nofima AS, N-1431 Ås, Norway; 2 TIK Centre for Technology, Innovation and Culture, University of Oslo, N-0317 Oslo, Norway

**Keywords:** Sustainability-oriented innovation, Food industry, Dynamic capabilities, Sustainability transition, Strategy, Incumbent

## Abstract

The urgency of sustainability transition requires large incumbents in the food industry to implement sustainability-oriented innovation (SOI). However, the high concentration of the food sector and the complexity of the sustainability concept make its understanding and overall transition challenging and slow. Incumbents would need to drive the transition by redesigning business models and practices and acquiring new competencies to integrate sustainability into their innovation strategy. This paper has a twofold aim: (I) analyzing the evolution of sustainability understanding over time and (II) evaluating the extent of dynamic capabilities of food incumbents to foster SOI. We developed an integrated theoretical framework combining the theory of dynamic capabilities with aspects of SOI and applied it to the case of the Norwegian food industry. We interviewed eight food incumbents and one food industry association, and we reviewed their annual and sustainability reports from 2016 till 2020. Key findings show a high strategic activity in SOI, as well as a notable and industry-wide ambiguity about what sustainability means in the food sector. Most companies reveal both an adaptive and expanding behavior implementing conscious sustainability-integrated product and process innovations. Most innovations are incremental without a radical modification of business models. Some exceptions have been detected resembling transformative changes. Clear initiatives of moving away from a linear supply chain to a more systematic approach are currently happening through food system collaborations.

**Supplementary Information:**

The online version contains supplementary material available at 10.1007/s43615-022-00234-1.

## Introduction

### Background and Research Gap

Addressing sustainability challenges is an urgent matter in the food system, as food production is linked to key environmental issues [1–3]. For example, it contributes for 26% to global greenhouse emissions, livestock represent 94% of mammal biomass (excluding humans) contributing to lose biodiversity, whilst agriculture uses 50% of global habitable land and is responsible for 78% of ocean and freshwater pollution [4].

Nevertheless, in the food system, the concentration of power in the hands of a few large, well-established firms—the incumbents—makes the transformation process slow and complex [5, 6]. Incumbents constitute the backbone of the food industry and could use their power to drive the sustainability transition by facilitating the diffusion of new technologies [7]. Thus, for an impactful sustainability transformation of the food system to take place within the next years, change must come from them. It is no longer sufficient for them to comply with environmental regulations or only incrementally innovate. Incumbents must consider sustainability as a concrete business opportunity [8, 9] rather than thinking of it as costly and time-wasting and limiting themselves to passively responding to regulatory requirements [10–12]. It is therefore expected that they redesign internal structures and processes, acquire new competencies, and increasingly invest in innovation by integrating sustainability into their strategy [13–16]. The 17 Sustainable Development Goals (SDGs) developed by United Nations in 2015 offer guidelines for action to firms for achieving business opportunities enabling them to improve their economic results with initiatives that add environmental and social value [8, 17, 18]. For example, in the agri-food system, firms can profit by opportunities such as implementing technologies to reduce food waste in the value chain, introducing digitalization in farms, investing in sustainable product innovations (e.g., compostable packaging, new foods which are affordable and nutritious), facilitating procurement from low-impact farms, using byproducts, and improving the circularity of the value chain [4, 17].

However, as SDGs are addressed globally, firms might have difficulties while building sustainable strategies. To this end, the UN Global Compact initiative has launched a program aimed at supporting firms in understanding and translating SDGs into practical actions which are relevant for their respective sectors [19]. Also, each country has published sustainability strategies with targets to be reached in different industries by 2030.

To respond to SDGs and country-specific sustainability strategies and contribute to a sustainable transition for the future, incumbents need to reduce their impact on the environment by developing sustainability-oriented innovation (SOI) [16, 20]. SOI encompasses the improved products, services, or business models providing benefits to the environment—compared to the alternatives—in addition to value created for companies and customers [13, 14, 21]. SOI is often multidisciplinary and requires competencies across different sectors [22]. Operationalizing SOI is an unfolding process which occurs over time; therefore, firms must possess dynamic capabilities [15, 23–26] enabling them to adapt and acquire the appropriate know-how and/or develop competencies to progressively restructure business models [27, 28]. Current organizational procedures must be shaped within and across the firm’s boundaries towards new patterns by involving value chain partners and industry stakeholders [14, 29, 30].

Incumbent firms in the food system are often resistant to change and hesitant to engage in new organizational practices and adopt new technologies [3, 22, 27, 31]. They struggle to develop the necessary flexibility to promptly react to the call for sustainability with innovation when they already have a rooted business. However, Turnheim and Sovacool [32] recently called for more research on the role of incumbents in the transition towards sustainability to provide a plurality of visions and analyze alternative perspectives of incumbents’ behaviors in front of a change, shifting away from the traditional literature that emphasizes their static tendencies.

So far, the need of dynamic capabilities for sustainability has been mostly explored in a theoretical context, unveiling the need for empirical investigation, especially at the micro-level [15, 33–35]. There is a lack of empirical studies exploring how companies engage in SOI and which dynamic capabilities are developed and deployed to this aim [24, 36, 37]. Furthermore, the food industry, having a traditionally lower level of R&D investment, has often been overlooked in previous research on dynamic capabilities [24].

Despite sustainability being high on the international policy agenda [38, 39], food incumbents have not developed a common understanding of the topic, and consequently integrate it differently in their strategy and across functions. The concept of sustainability is indeed composite and defined by several notions that have evolved over time [40]—from pure compliance to corporate social responsibility (CSR) to the triple bottom line [41] and to conformity with the UN SDGs [42]—creating a further complication.

Finally, research on the role of incumbents in the sustainability transition and how they integrate it in their innovation strategy is scarce in the food industry. Literature searches on both Scopus and Web of Science using the syntax « “sustainab*” AND “incumbent” AND “food” AND “innov*”» in the title, abstract, and keywords returned only 20 and 37 articles, respectively.^1^ Among these, only eight concern sustainability-oriented strategies developed by food incumbents. It is thus of pivotal importance to explore these topics.

We analyzed a case study of the food industry in Norway, the largest mainland industry, contributing 40% of the value creation [43]. The sector consists of a few large (incumbent) and many small firms. It is highly concentrated, particularly at the wholesale but also at the retail level. Agri-food value chains are heavily influenced by the primary market regulation provisions of agricultural policies, including target prices and production quotas (for dairy) and import tariffs and quotas [44].

In Norway, the debate about sustainability understanding is very active, and firms are working to comply with it and integrate sustainability in their strategy [45]. However, a recent survey on the status of sustainability across various sectors, including food, showed that knowledge is still poorly disseminated across organizational functions. Less than 30% of respondents had a sufficient level of competence among their employees to work strategically with sustainability, and its implementation is still quite slow [46]. However, the intense political focus on sustainability in the food system [48–491] has led to the formation and engagement in sector-wide networks. Indeed, the Norwegian sector agreement aims at reducing food waste by 30% by 2025 [49], and a sector-agreed road map for circular plastic packaging is in place [50]. A voluntary sector agreement to reduce emissions by 5-million-ton CO_2_- equivalents in the period 2021–2030 is in place [51] as well as a strategy document called “Food Nation Norway” that combines aspects of sustainability, innovation, economic growth, and public health in an SDG-based action plan towards 2030 [52]. The development of new technologies to reduce climate gas emission during food production is in focus in the Norwegian climate plan 2021–2030 [53]. Overall, sustainability discussions in the food system are complex due to its multi-stakeholder nature, intertwined structures, Norway’s difficult geography, and the important role of policymakers and consumers [19, 54].

Against this background, we aim to explore how the call for sustainability is shaping innovation in the Norwegian food industry by answering the following research questions:

1. How is sustainability understood and defined by incumbent food companies in Norway?
2. To what extent have incumbent Norwegian food companies developed dynamic capabilities facilitating the development of SOI?

The remainder of the paper is structured as follows. The “Incumbents` Engagement in Sustainability” section highlights the relevance of the topic of incumbents’ role in the scientific debate on sustainability transition. The “Theoretical Perspectives” section reviews the theoretical perspectives on how sustainability can shape firm innovation strategy; we propose an integrated framework to analyze our case. The “Materials and Methods” section presents the methodology and describes the sample of firms interviewed. In the “Results and Discussion” section, we report and discuss the results: the “Strategic Shift in Focus from CSR to Sustainability Accountability” section answers the first research question, and the “From Dynamic Capabilities to SOI” section answers the second research question with an analysis of case findings through our theoretical framework. Finally, the “Conclusion and Implications” section summarizes conclusions.

### Incumbents’ Engagement in Sustainability

Incumbents are fundamental in shaping the food system’s transformation towards sustainability. A change at the micro level triggered by established food firms is needed to make a visible impact at a systemic level. Therefore, incumbents must modify their organization or processes and remodel their long-term strategy including periodical finetuning of goals to adjust them to sustainability transition’s challenges [20, 24, 55]. However, innovations with a significant positive impact on sustainability are often radical and disruptive [31], shaking the stability of incumbents’ strategies and conflicting with their interests. Incumbents can be alarmed by SOI, as these can boost competition from new entrants, make established core competences obsolete, or even intensify the risk of implementation-related technical issues [31].

A dominant stream of literature depicts incumbents as unable or unwilling to modify their strategies to engage in sustainability transitions [5, 56–59]. In his recent article, Béné [5] analyzed the difficulties of making a substantial change in the food system. The article focused on the role of multinationals and large firms that polarize the power of the entire system, from the input supply to food production and distribution. The market power being so concentrated in the hands of a few, there is no interest in engaging towards more sustainable practices [5, 60, 61]. Béné [5] strongly affirmed this: “instead of being a source of innovations, the largest agri-food corporations direct most of their resources at protecting their own assets and investments, resisting changes, and preventing any new or disruptive innovation from entering the sector, thus de facto contributing (purposively) to lock the system in its current status quo.” Likewise, Smink et al. [31] focused on incumbents’ institutional strategies and found that firms are active and able in shaping policies and lobbying, mostly underscoring the drawbacks of certain innovations to defend their interests. Incumbents are indeed able to influence policy by exerting pressure on specific reforms or standards or shaping political campaigns and information [62].

Another reason for incumbents to resist change and adopting defensive strategies seems to be associated with preventing new technologies from disrupting the market and reducing the competition from new entrants. Old business models can make incumbents more vulnerable and force them to use their power to hamper any change threatening them [63–65]. In the agri-food sector, geographical factors inhibit sustainability integration. Especially for those firms strictly dependent on primary agricultural production, shifting to more sustainable production processes is not always feasible in the short term, as farmers are dependent on soil and climate characteristics, biodiversity conservation, and other local-specific issues [66]. Since the integration of sustainable practices in the agri-food sector is very particular, each stakeholder needs to develop new approaches that fit its region or country, which can sometimes lead to slowness or resistance to change.

However, a second bundle of studies describe opposite results concerning incumbents’ potential to stretch out of their comfort zone and willingness to engage in transformative sustainability practices. The studies show different responses to the need for change according to the industry setting, firm-specific capabilities, and type of challenge to tackle [32]. Although incumbents are often challenged by new entrants, they also possess the resources and capacities to react and behave like “fast followers” by adapting their innovation processes or establishing ventures with some of the challenging start-ups, especially when the innovation is disruptive [67]. Overall, these studies, carried out in different industries and mainly on a case-study basis, demonstrate that incumbents can leverage and exploit their established resources and competencies to capture value from innovations from outside their boundaries. The capacity for ambidexterity and redesigning extant strategies and processes associated with SOI in multiple ways has been observed [67–69]. Incumbents can perceive the value of implementing SOI for achieving a better competitive position, for shaping the institutional change, or for valorizing their resources into something incrementally or radically new [32]. Several studies have analyzed the role of complementary capabilities for incumbents to implement SOI [70]. For example, they can implement a new technology by reconfiguring their processes and/or organization and complement it with their already established competency for uptake and commercialization. Complementary capabilities have also been discussed as survival enablers for incumbents in front of a technological change. Incumbents engage in open innovation to complement the missing knowledge and competencies associated with an emerging and disruptive sustainable technology [71].

Focusing on the food industry, Strøm-Andersen [24] found that dynamic capabilities are key for adapting businesses to new opportunities and generating profit out of them in a long-term perspective. They are pivotal for competitive advantage and for implementing innovations. Even in a sector traditionally presented as low-tech as the food industry, incumbents can be proactive and acquire knowledge from other sectors in an open setting, exploiting their networks and core competencies [24]. Incumbents with strong dynamic capabilities thus have a better likelihood of sustaining a transition [72]. Saari et al. [73], who analyzed both incumbent and start-up food firms contributing to sustainability by entering the plant-based market, found similar results. Interestingly, they found that incumbents were proactive in adapting their product portfolio and strategy by introducing new ingredients and complementing them with their extant technology. They coexist with new entrants who developed new products and implemented new technologies. Therefore, institutional change does not only hinge on young firms challenging incumbents or on huge market disruptions but needs a heterogeneous set of actors [73].

## Theoretical Perspectives

### Sustainability-Oriented Innovation

SOI encompasses “making intentional changes to an organization’s philosophy and values, as well as to its products, processes, or practices, to serve the specific purpose of creating and realizing social and environmental value in addition to economic returns” [14]. The concept of SOI has evolved by not only limiting its scope to produce benefits for the environment and society but also to generate profits for the firm. Therefore, a firm that integrates sustainability into its innovation strategy by developing SOI can positively impact the environment and society while maintaining or even enhancing its competitive advantage in the market [13, 14, 16, 74]. Moreover, management of SOI is not only limited to the product’s use but concerns the whole lifecycle, from raw materials to commercialization and beyond [75], to detect any sustainability issue along the value chain. Also, in recent literature, SOI has often been associated with circular business models as a way to operationalize it [40, 76].

The integration of sustainability within a firm’s innovation strategy can be either a top-down or bottom-up effort. In the first case, sustainability is considered in the strategic management plan as a key element of the vision, goals, and operations of the firm; in the latter case, SOI is generated by employees’ intrapreneurial initiatives [14]. Other internal drivers of SOI relate to specific know-how and competencies and technological and managerial skills, whereas external drivers include cooperation and partnerships with other stakeholders like academic and private research institutes, market pull, and regulatory push effects [77].

Differently from the various denominations connecting sustainability with innovation (e.g., sustainable innovation, CSR-driven innovation, and sustainability-driven innovation) offered by the scientific literature so far, SOI implies that innovation is an evolutionary process towards sustainability that needs a management effort and aims for “relative improvements in comparison to a prior or other entity” [13]. Previous research proposes that SOI includes different implementation stages [35]. Adams et al. [14] carried out a systematic literature review on the topic culminating with a comprehensive framework based on three evolutionary phases along which sustainability can be integrated into the main categories of innovation management (strategy, innovation process, learning, linkages, innovative organization). SOI activities can thus be organized along the following stages: operational optimization (“doing more with less”), organizational transformation (“doing good by doing new things”), and system building (“doing good by doing things with others”) [14]. *Operational optimization* characterizes a firm that integrates sustainability by exploiting its current innovation activities [14]. It has a reactive behavior aimed at complying with immediate environmental needs and regulations, and only engages in incremental innovations, prioritizing its own efficiency and sticking to operational excellence [78]. *Organizational transformation* implies a firm changes its mindset by expanding its focus on society as well. Instead of being concerned with reducing its negative impact, it shifts towards producing a positive one by implementing new activities. The sustainability integration within the organization is still internally oriented, although with a reinterpretation of the impact that each function increasingly accounts for short-range stakeholders. The internal structure experiences a redefinition of relationships and a better valorization of people. Lastly, *system building* implies a huge step toward radical change. Here the firm understands that sustainability is not only limited to an individual effort; it must be understood globally to return a significant effect. Although the sustainability input can start within the organization, the collaboration with and inclusion of relevant stakeholders of the value chain, system, and society at large becomes key to creating sustainable value through integrated cooperation. The aim is to develop radical and “system-shaping” innovations [14]. Therefore, exploring other domains and searching beyond usual partners is pivotal to complement knowledge and catch new opportunities [79]. For example, incumbents may innovate towards more effective circular practices like maximizing material and energy efficiency with new loops (e.g., recycling, reusing, reducing resource use), delivering functionality instead of ownership (e.g., leasing, pay-for-use), or engaging consumers in co-creative alternative consumption platforms (e.g., sharing economy), all having implications on their governance and contributing to the development of circular business models [76].

After the comprehensive literature review on the stages of SOI carried out by Adams et al. [14], which is still widely acknowledged as target reference and highly cited,^2^ research on SOI has strengthened and evolved showing the relevance of the topic. Different research streams are focusing on and analyzing specific issues aimed at understanding and facilitating SOI implementation. Some relevant issues are, for example, impact of SOI on organizational performance [80, 81]; alliance proactiveness, cooperation networks, and supply chain relationships as necessary to pursue SOI in an open innovation setting [79, 82, 83]; the role of different stakeholders involved in SOI [84]; information technology as SOI facilitator [85]; design thinking as tool for SOI development [86–88]; the role of individuals’ SOI narratives on the adoption of nature-inspired innovations [89, 90]; and the potential of blockchain to contribute as SOI [91].

### Product, Process, and Organizational SOI

Regardless of the stage of SOI and the depth of sustainability integration, SOI can be developed through product, process, and/or organizational innovation [13, 77]. Product SOI ranges from bare improvements to the ideation and production of goods with a reduced environmental impact. Sustainable product development seeks to contribute to sustainability while also keeping the firm competitive through a sustainability-integrated strategy [92]. The concept of eco-design comprehensively underlies several examples of sustainability integration in product innovation: reduced energy consumption, use of recycled materials, eco-labeling, packaging reduction, reusable packaging, use of fair-trade and organic raw materials, refurbishing properties, and extension of durability. Being characterized by a life-cycle perspective, some of the abovementioned examples can also imply a process or an organizational innovation, though the product dimension prevails [13]. Product SOI helps firms stay competitive through a better corporate image and operational and financial performance, the creation of new market segments (in the case of radical innovation), and enhanced product quality [16, 93–95].

Process SOI includes restructuring operations within the organization and along the value chain to achieve eco-efficiency, namely reducing environmental impact while also being economically efficient [96]. Examples include energy saving and substitution of obsolete equipment. More recently, the concept of cleaner production has been introduced as an overarching term to identify how a firm can innovate the production process towards sustainability. This has been associated with the implementation of green technologies, replacement of fossil resources with bio-based ones, good housekeeping, waste management, and byproduct valorization [13, 14, 22]. Logistics also play a relevant role in making processes more sustainable through the change of transportation modes, distribution channels, and improved fleet management [13].

Organizational SOI entails redesigning the firm routines, work organization, and internal procedures towards an enhanced social and environmental management system and, in turn, effective business models, which can span towards a circular design [13, 28, 97]. Although it is often a prerequisite for operating process innovation, it is more focused on people and work responsibilities, integrating a social dimension. Organizational SOI allows to implement sustainability in the strategy by changing the code of conduct and sustainability vision, developing training for employees for better engagement in CSR activities, and innovating the supply chain and stakeholder management towards circular process loops [13]. Internally, the organization can implement changes, such as creating new departments or cross-functional units dedicated to environmental management or hiring new human resources with specific competencies in sustainability management. The modification of the pool of stakeholders to cooperate with or the inclusion of new ones constitute other approaches to innovate towards sustainability and circular economy [76, 79, 83]. Establishing contacts with governmental institutions improves the dissemination of knowledge about new environmental challenges and might ensure financial support. Research institutions provide often lacking expertise and can assist firms in managing the complexity of SOI, whereas unconventional actors (i.e., influencers, visionary entrepreneurs, local communities) can trigger new ideas and radical innovations [98, 99]. These secondary stakeholders have a proactive role in supporting the formation of new capabilities and offer opportunities for value co-creation as SOI “stimulators” and/or “initiators” and can help incumbents to innovate their established business [84]. Concerning the existing relationships along the value chains, strengthening supplier–buyer cooperation motivates suppliers towards SOI and allows buyers to have better control backward in the chain [13, 100]. However, suppliers and buyers, having a direct interest in the current business model, may represent a challenge when it comes to innovate, because it implies extra efforts to change their current operations as well [84].

### Dynamic Capabilities

Dynamic capabilities describe a firm’s capacity to adapt its core competencies and organizational routines in response to fast-changing environments [101]. Translating this concept to the actual context, incumbents face the urgency to respond to the transition of the current economy towards sustainability and are urged to consolidate social and environmental goals with economic ones. Dynamic capabilities are thus pivotal for firms to foster sustainable practices [15, 23, 24, 26, 102]. The well-acknowledged framework from Teece [103] introduces three types of dynamic capabilities: sensing, seizing, and reconfiguring. Sensing capabilities allow the firm to learn and identify opportunities in the firm environment [101], either internally or externally [104]. Internally, firms can promptly share knowledge and diffuse it across functions. Externally, they can monitor external threats; explore markets, consumer preferences, and trends; listen to suppliers; search for potential partnerships; and heed regulatory or policy changes. Through seizing capabilities, firms can capture value from recognized opportunities by developing and introducing new products and services [15]. Firms need to mobilize resources to engage in R&D, prototyping, and acquiring knowledge through research collaborations and partnerships. They also can participate in regulatory and policy processes and political lobbying and agenda-crafting.

Reconfiguring capabilities describe the ability to reorganize and restructure the organization for continuous renewal, including company culture, knowledge management, and co-specialization that supports innovation for sustainability. Firms are more open to innovate and cooperate with a wider set of partners [26]. Also, co-specialization occurs either when a firm combines in-house skills in different business units—as they deliver higher value together than separated—or when a firm integrates its internal skills with those of external partners through mergers, joint ventures, or other collaboration [103].

### Integrated Conceptual Framework

SOI implies that firms must move their spotlight from achieving a competitive advantage solely to achieving it with a reduced impact on the environment and society [26, 105]. The need for reshaping the innovation strategy towards sustainability asks firms to develop dynamic capabilities [26, 105]. However, due to the complex and fast-evolutionary character of SOI, dynamic capabilities must “involve more comprehensive and socially complex innovation management, production, and manufacturing processes […] cross-stakeholder management and engagement and system-thinking” [35].

So far, the connection between dynamic capabilities and SOI has been approached in a disjointed way. Quite recently, however, Inigo and Albareda [35] conducted a systematic review followed by a multiple case study and delivered a three-level categorization of dynamic capabilities aimed at SOI: adaptive, expanding, and transforming*.*

*Adaptive capabilities* imply a continuous resource modification to enhance through learning individual and organizational competencies and knowledge towards sustainability [35]. They correspond to sensing capabilities, which also serve to calibrate resource use according to potential opportunities [15].

*Expanding capabilities* express the implementation of novel sustainable practices and the development of new organizational capacities within the firm. Firms seize opportunities by diffusing SOI to the market, establishing new networks to integrate sustainability into innovation, adopting clean technologies, and acquiring external knowledge.

*Transforming capabilities* represent the highest-order level. They are manifested when a firm has a systemic view and reconfigures its business model entirely by increasingly involving external partners and stakeholders. Economic, social, and environmental values are created jointly with the intention of generating a resilient system through advanced learning processes with stakeholders going beyond firm and industry boundaries.

**Fig. 1:**
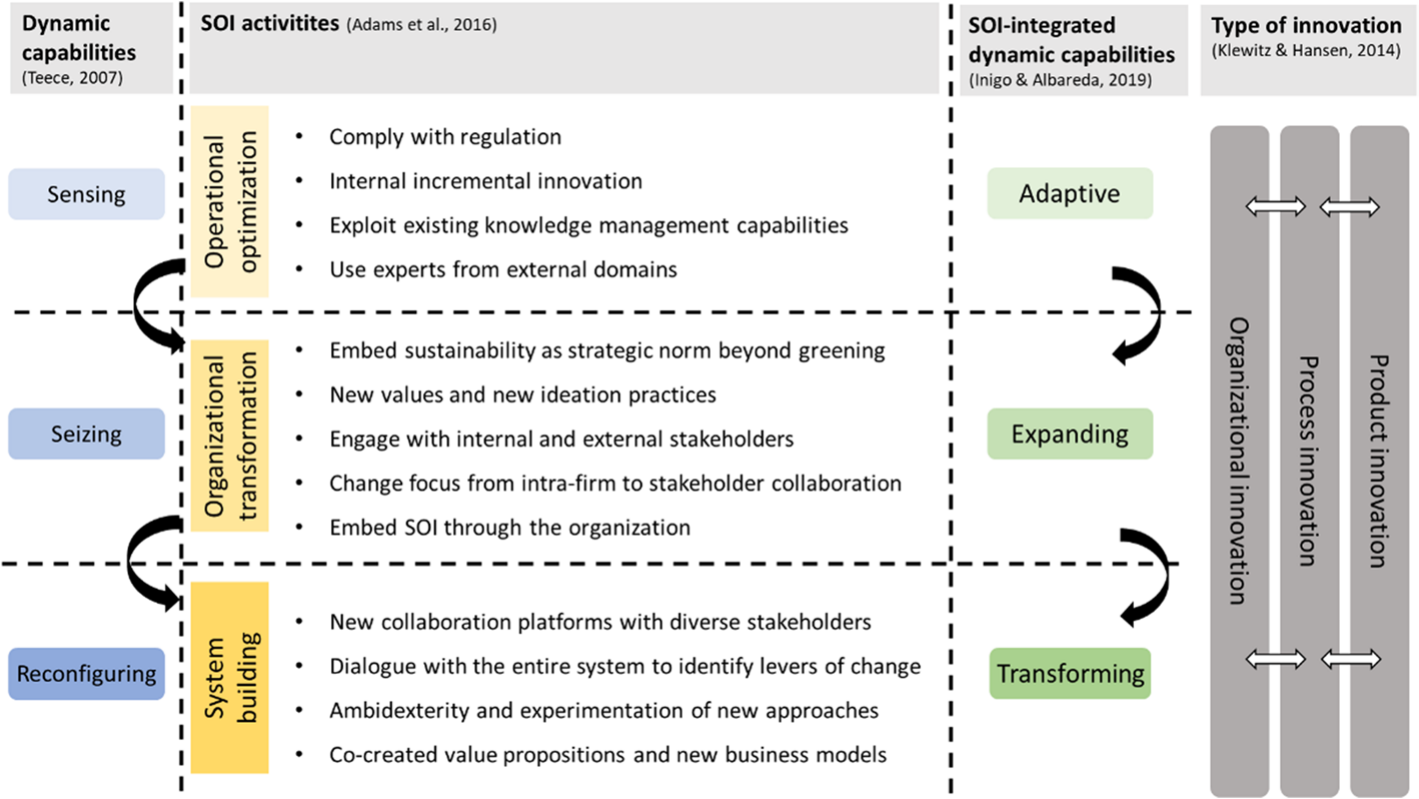
Conceptual framework integrating dynamic capabilities, SOI, innovation types, and SOI-integrated dynamic capabilities [13, 14, 35, 102]

This framework was applied to seven industrial sectors but excluded the food industry. Thus, we focused on the food system and examine to what extent food incumbents in Norway have developed SOI-integrated dynamic capabilities. We based our analysis on a conceptual framework (Fig. 1) derived from the abovementioned theoretical frameworks of dynamic capabilities (the “Dynamic Capabilities” section), SOI (the “Sustainability-Oriented Innovation” section), SOI-integrated dynamic capabilities (the “Integrated Conceptual Framework” section), and type of innovation (the “Product, Process, and Organizational SOI” section).

## Materials and Methods

A multiple-case-study approach was applied based upon the approaches’ applicability in inquiring about a real-life phenomenon in a dynamic context [106]. According to established case study research standards, the ideal number of cases is between 4 and 10 units of analysis [107]. The case study approach is very feasible to study a real live phenomenon in a dynamic environment (SOI dynamic capabilities). We selected ten food companies among the largest in Norway (eight of them rank within the 40 largest companies) [108] to represent actors along the value chain (primary food producers, food manufacturers, and retailers) and across different national subsectors, such as dairy, meat, cereal, ready-to-eat, and fish/seafood producers (Table 1). For each of these companies, we reviewed documentary data such as strategy documents, annual reports, and sustainability reports from 2016 to 2020. We created a database of the key sustainability issues for each company and a timeline of their emergence, as well as tracking product launches that carry a sustainability notion or claim.

**Table 1:** Overview of case companies and interviews

Supply chain position	Company acronym	Company information	Interview informants
Primary production and processing	PP1	Dairy ~ 5,000 employees	Head of research-based innovation and senior brand manager
Primary production and processing	PP2	Meat ~ 5,000 employees	Director of R&D and innovation and EVP of communication, sustainability, CSR^*^
Primary production and processing	PP3	Fruits and vegetables ~ 3,000 employees	Director of sustainability and innovation
Primary production and processing	PP4	Seafood ~ 5,000 employees High export rate	Head of sustainability and quality and head of product development
Processing	P1	Cereals ~ 150 employees	Director of innovation
Processing	P2	Foods and consumer goods ~ 18,000 employees	Head of sustainability and director of product development
Processing	P3	Foods ~ 400 employees	Head of creative innovation and Head of sustainability
Distribution and retail	DR1	Retailer ~ 27,000 employees	Head of CSR and sustainability^*^
Distribution and retail	DR2	Retailer ~ 4,200 employees	Not interviewed
Distribution and retail	DR3	Retailer ~ 40,000 employees	Not interviewed
Food industry association	IA1	Industry association ~ 1,600 members	Chief adviser of food policy

^*^No longer employed at the respective company at the time of manuscript submission/publication

We followed up the desktop study with expert interviews with industry actors to form a coherent understanding of the companies’ strategies. Eight firms agreed to be interviewed. The 14 informants consisted of managers responsible for environment, CSR, or sustainability issues, as well as managers responsible for innovation and product development. Interviewing two persons from each case company representing both sustainability and innovation/product development further contributed to the richness of insights and data by offering a holistic view across functions. We conducted one supplementary background interview with an expert from a food industry association. Interviews were performed in the period April–October 2020 and lasted between 30 and 80 min. All interviews were conducted digitally using Microsoft Teams, respecting COVID-19 restrictions. Whenever possible, two people from the same firm were interviewed together to gauge synergy or disparity between innovation and sustainability work. The interviews were recorded and transcribed verbatim. For the data analysis process, a thematic approach was used, providing a systematic and stepwise procedure for synthesizing data from the interview narratives combining thematic analysis [109] and the approach from Gioia [110]. First, two researchers manually coded the transcripts independently, and in a second step, all three authors actively explored the coded data to find similarities and overlap between the codes to develop themes and identify deeper meaning and relational structures. Theoretical insights informed the case and provided the analytical lens through which data was interpreted. In the last abductive step of the analysis [110], we used our theoretical framework as a diagnostic tool, to explain if, how, and why sustainability is integrated into firms’ innovation strategy to address the goal of food system transformation.

The authors also attended key industry events and network activities that focused on sustainability, i.e., S-Food/S-HUB Norway—a network of companies that aim to facilitate sustainable innovations, Matfloken, a multi-actor open innovation project across the supply chain tackling SOIs. As such, we drew on extensive contextual knowledge.

## Results and Discussion

### Strategic Shift in Focus from CSR to Sustainability Accountability

Food system sustainability is complex, and notable industry-wide ambiguity persists about what sustainability means in the (Norwegian) food sector [45]. We found that the companies interviewed are sensitive to the fact that food production is complex and carries some contradictions in terms of sustainability (*“The goal is to provide healthy food and good nutrition for people. Food production is a means to achieve this, while carbon footprint is an inevitable consequence of food production. Therefore, one must always see the undesirable consequences (environmental impact) considering the objective (good nutrition), in other words, how eco-efficient food production is in relation to the nutritional value.”—PP1*).

All companies in the case have used the UN SDGs [42] as their guiding standard, and many also mention the triple bottom line “people, planet, profit” [41] or the Brundtland Commission [111]. These goals are broad and can encompass everything. We observed a purposeful selection of SDGs linked to existing strategies and capabilities. One informant (P3) explained that *“We use the SDGs, you know, those icons. We’ve chosen 2, 3, 8, and 12, as they’re the most important ones, and then we also have 13, 14, and 15.”* All informants answered with similar statements.

We also found that the incumbents’ understanding of sustainability is broad. One sustainability manager (DR1) explained the job as *“I work across all areas of our value chain with sustainability and social responsibility, in which we include environment, climate, health, social inclusion, and responsible trade.”* The same informant also said, *“We are in the midst of a new strategy, and it is obvious that climate and environment is one part of it, but it is not only climate but also natural resources, biodiversity, use of oceans and land use, and everything. We produce food. It’s also health, our consumers’ health, working conditions, diversity, and social inclusion.”*

Using such a broad definition—which includes diverse aspects like workers’ rights, health and safety, food safety, and public health—could potentially “dilute” the concept of sustainability. After having reviewed both the companies’ annual and sustainability reports, however, we found much more nuanced operationalized goals and targets. Thus, we interpret vague and broad definitions not as an excuse for picking easy targets but as a reflection of the complexity of the food system. This interpretation is also backed up by interview data in statements such as the following: *“Our sustainability strategy is health; healthy food is important, as is safe food, and its sustainable sourcing of raw materials, from farm to fork and everything in between … And there is climate and environment … we have always cared about people and community … health has been important to us for the past 20 years, while the planet and CO*_*2*_* and such has become more and more important recently as we’ve seen the threat to the planet increase”—P2.*

**Fig. 2:**
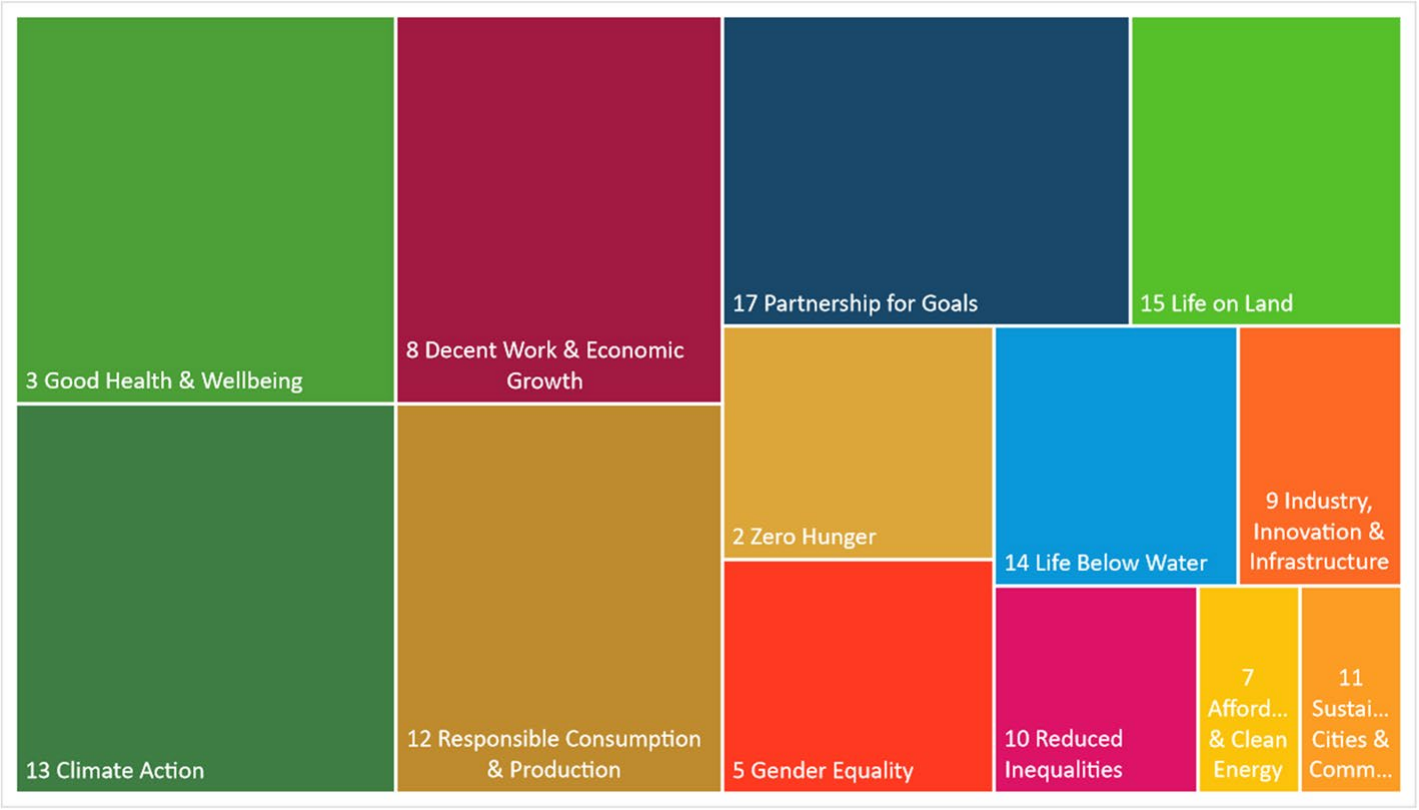
Tree-map of the SDG goals in focus for the incumbent Norwegian food firms in this study

While all incumbents mentioned the SDGs, seven out of nine have clearly spelled out the individual goals in their strategy and key performance indicators (KPIs). The tree-map chart in Fig. 2 represents the respective SDGs most in focus for the Norwegian food incumbents in this study: Climate Action (SDG13), Good Health and Wellbeing (SDG3), Responsible Consumption and Production (SDG12), Decent Work and Economic Growth (SDG8), and Partnership for the Goals (SDG17).

We also found that organizations are moving from what was previously termed social accountability and CSR towards integrating climate, natural environment, and sustainable innovation in a systematic way, as illustrated by the following quote: *“The word sustainability has not been used, no. It has come up later. We have been very familiar with everything we talk about but not as a sustainability strategy”—P3.* The recruitment of new managers with specific skills for sustainability within extant organization units, as exposed in the next section, also evinces this trend.

Furthermore, the document analysis revealed a more differentiated and detailed picture regarding visions, goals, and KPIs for sustainability. All incumbents have integrated sustainability in their vision and goals, confirming a high strategic importance and focus on SOI [14]. Some exemplary vision statements included *“We want to be one of Norway’s most attractive and sustainable food companies”—PP2*; *“We want to become the most profitable global supplier of sustainable quality seafood”—PP4; and “We want to give the customer a healthier and more sustainable everyday life”—DR1.*

**Fig. 3:**
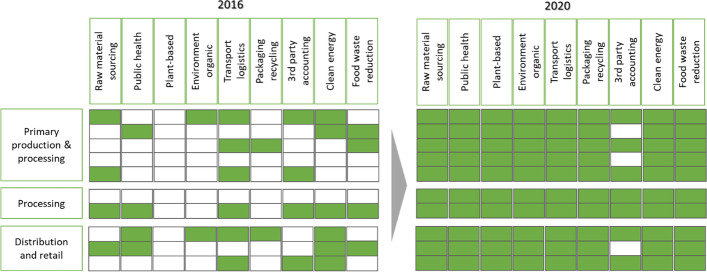
Elements of sustainability addressed in companies` strategy (one line represents one company; headings derived from qualitative content analysis of annual and sustainability reports)

Figure 3 provides an overview of sustainability elements addressed in companies’ strategies and shows the change from 2016 to 2020. This image confirms SOI as an evolutionary process with different implementation stages [13, 35], moving from sparce initiatives at some firms in 2016 to a broad engagement and high activity for all incumbents in 2020. Organizations operationalize SOI according to the areas they can impact in line with a formal materiality analysis [112]. For two companies represented in Fig. 3, we did not find any official documents or reports that contained sustainability relevant information in 2016 (white cells). From the interviews, it became obvious that does not necessarily mean that these companies did not work with aspects of sustainability; however, they did not publicly share that information. A change in focus has happened across all the incumbents and in 2020 most of them work with all elements of sustainability.

### From Dynamic Capabilities to SOI

Concerning the second research question, we found that all the respondents started with an adaptive firm behavior to respond to sustainability and then progressed through expanding their capabilities further. Only a few have a systemic view. In general, firm-internally, SOI was found to be a top-down effort and is first anchored in strategy planning and formulation and then operationalized in single business units and functions [14]. Innovation processes are first adapted to the company’s sustainability strategy and goals. However, organizational and cultural change and new capabilities are recognized as needed but are simultaneously challenging to define and implement. A dedicated sustainability function was established in recent years in all companies to formalize and operationalize sustainability work. All interviewed companies highlighted that they have a good starting point in terms of sustainability, as they use Norwegian raw materials, which they consider inherently more sustainable versus imported goods due to the intensive agriculture, lower use of fertilizers, and shorter transport.

Our findings as related to the integrated conceptual framework from Fig. 1 are summarized in Table 2 below. The detailed findings from the analysis of the interviews are structured according to the SOI-integrated dynamic capabilities and explained in the paragraphs following Table 2.

**Table 2:** SOI activities and SOI-integrated dynamic capabilities of Norwegian incumbent food firms

Dynamic capabilities [103]	SOI activities of Norwegian incumbent food firms	SOI-integrated dyn. capabilities [35]	Type of innovation [13]		
	**Operational optimization**		Organizational	Process	Product
Sensing	- Removal of palm oil (consumer push)	Adaptive			
	- Trend scouting for sustainable innovations abroad				
	- Use of experts from external domains				
	- Policy monitoring				
	- Cost and efficiency focus				
	**Organizational transformation**				
Seizing	*New values and ideation practices for SOI*	Expanding			
	- Implementation of renewable energy solutions (solar, biogas, electricity, hydrogen)				
	- Development and launch of new plant-based products				
	- Using existing technologies for new innovations				
	- Tapping into new markets				
	- Informing consumers about sustainability				
	- Recycling and reduction of plastic				
	- Food waste reduction				
	- Value capture through utilization of waste/by-products				
	- Health as a dimension of sustainability				
	*Embedding new organizational practices and norms*				
	- Establishment of dedicated sustainability function/role				
	- Establishing new norms, sustainability strategy and goals				
	- Supply chain integration in SOI				
	*Increased engagement with internal and external stakeholders*				
	- Engagement in policy making activities				
	- Participation & commitment to public–private partnerships				
	- Sector wide open innovation efforts				
	**System building**				
Reconfiguring	- Food system thinking and engagement	Transforming			
	- New collaborative platforms				
	- Increased ambidexterity				
	- Research and investment in radically new technologies				
	- Developing and implementing circular business models				
	- Regenerative production (negative footprint aspiration)				

#### Adaptive Firm Behavior

For the early 2000s, SOI in the Norwegian food industry was described as operational optimization and a focus on regulatory compliance. While this is an ongoing effort at the core of many businesses, regulatory compliance was not merely mentioned as a strategy towards sustainability but as a housekeeping factor within the incumbent food firms.

All companies considered important the role of consumers’ changing shopping and eating behaviors linked to their demand for more sustainability, which we interpret as a market-pull effect to SOI, as described by Jarmai [77]. Customers and shareholders were strong external drivers for sustainability for all interviewed companies. They have been able to modify their resources to comply with sustainability requirements derived from the market [35]. As an example, palm oil was completely removed from all products after strong consumer reactions to a documentary shown on Norwegian TV in 2012 (P2, P3). This is illustrated by the following quotes: *“If consumers start pushing, the industry must take action—and we can of course be someone who can go first. We see that our customers are also beginning to have sustainability goals, and, if we have to be a relevant supplier for our customers, then we must be aware of that”—P1; *and* “There will be a generation that has [sustainability] as a hygiene factor and that has it in attitudes and values ​​from a young age”—DR1.*

Trend-scouting was identified as sensing behavior in all the interviewed firms. Companies actively scout for information on new products and consumer needs through both their marketing and R&D/product development (PD) functions. A typical behavior is to learn from experiences abroad and implement them in the Norwegian market; *“we follow closely what kind of innovations are happening around the world that are related to sustainability and try to take [them] to Norway”—PP2.* Some of the companies benefit from experts from external domains, and research collaboration facilitates the adaptation of current practices towards sustainability. For example, the EU “Farm to Fork” strategy [113]—with cornerstones of food loss and waste prevention, sustainable food production, distribution, and consumption—was mentioned several times as an interesting development to follow. This reflects a tendency to acquire knowledge not only about market trends but also policy and overall recommendations to play an active role in the food system`s transformation and adapt capabilities accordingly.

#### Expanding Firm Behavior

Intense efforts to implement a new strategic norm were identified for all interviewed firms. As described in the “Strategic Shift in Focus from CSR to Sustainability Accountability” section, the implementation, operationalization, and definition of specific goals in companies’ sustainability strategy in recent years are synonymous with organizational transformation.

##### New Values and Ideation Practices for SOI

We found that many incumbent food firms view SOI in the internal value chain is seen as a “housekeeping factor.” Efficiency improvements, focus on operational excellence [78], and cost reduction have been historically described as a core adaptive SOI activity for the incumbents. As an extension of operational optimization, organizational transformation towards cleaner production and an overall “greener” value chain was observed. Several companies strategically implemented process SOI focusing on renewable energy solutions (e.g., solar power, biogas) and energy-savings-enabling eco-efficiency (PP1, PP2, PP4, P2) [13, 16]. Electrification of transport and using hydrogen fuel are other observed actions to reduce CO_2_ emissions (PP4, PP1) [13]. We also extracted specific SOIs that cover known theoretical dimensions and are highly perceived by the consumer in the form of process SOIs.

Five companies out of nine developed and launched new plant-based products (PP1, PP2, P1, P2, P3), focusing on enlarging their portfolio towards products with reduced environmental footprints [77] and integrating new ingredients with existing technologies [73]. The latter exemplifies how incumbents valorize their available resources to drive innovation in new areas [32], enabling them to implement SOI while maintaining their competitive position. For two of the incumbents (PP1, PP2), this is also a strategy to compensate for a decline in animal-based sales, coexisting with new entrants by sustainably innovating instead of hindering development [73]. This SOI-integrated dynamic capabilities enable incumbents to adapt their business to new market opportunities [24].

Moreover, other incremental product innovation initiatives have been implemented, such as the introduction of environmental footprint labelling or sustainability indicators (P2, PP3). They aim to reduce information asymmetry between supply and demand by providing information to consumers and enabling conscious purchasing choices. Eco-labelling and indicators raise consumer sustainability awareness while enhancing the company’s image [93] and enable consumers to perceive SOI. Therefore, they also work as differentiation means [13]. Several SOIs also reduce environmental footprint and make a positive environmental impact, either through recycling and decreasing plastic use or shortening hauls for transport [13]. The companies interviewed are engaged in intensifying the use of recyclable and fiber-based packaging, as well as increasingly shifting their sourcing towards Norwegian and local raw materials, showing that they are expanding their competencies and activities towards a more responsible behavior [77]. Waste reduction was also mentioned by several interviewed companies as an optimization effort leading to more sustainability in line with Hermundsdottir and Aspelund [16].

Strong collaboration with R&D organizations and other firms in supporting sectors is necessary to facilitate these SOI activities and provide external competencies and knowledge as well as trigger new ideas in an open innovation setting [71, 98]. Despite these expanding SOI activities deep roots within the organization, they show the company’s intention to produce a positive environmental and social impact and to act for transformation instead of passively adapting to current practices [14]. Thus, “sustainability is no longer regarded as an add-on, but rather is/becomes embedded as a cultural and strategic norm” [14].

Besides SOI initiatives aimed at a more responsible production and consumption (SDG #12) and contributing to climate action (SDG #13), we also observed companies’ engagement in the research and implementation of nutritional improvements in products, such as reduction of sugar, salt, and saturated fats and increased dietary fiber content, all operationalizing the goal “Good Health and Wellbeing” (SDG #3). Health as a dimension of sustainability has a long history in the Norwegian food industry. A clear link between diet, health, and sustainability exists, as healthier diets are often more sustainable, and the adherence to dietary recommendations will benefit both health and the environment [114]. A broad range of SOI activities around health have been identified in our interviews—for example, the introduction of plant-based foods and the development of products low in calories. Moreover, food companies are engaged in many active public–private partnerships and sub-sector initiatives focused on improving public health through changes to individual products or product groups (e.g., the Salt Partnership,^3^ sugar reduction, reduction of saturated fat, bread-coarseness scale, new national dietary guidelines^4^).

##### Increased Engagement with Internal and External Stakeholders

Strong engagement with external stakeholders aimed at making the whole value chain more sustainable was identified, taking different forms depending on the value chain position of the respective firm. The vertically integrated companies focus on improving their primary production towards sustainable practices (i.e., biogas from cow manure, genetic breeding for reduced saturated fat, organic fertilizers). Others choose to trigger suppliers in collaborating in SOI to engage them in greener practices. This would establish high and demanding requirements upstream in the value chain, thus “enforcing” sustainability from the start of the product lifecycle, as illustrated by the following quote: *“[Sustainability] has also become a more important part of the procurement negotiations in recent years”—P2.* This organizational SOI confirms that strengthening supplier–buyer relationships can drive SOI [100]. This is also true for downstream value chain relationships. We found SOI-expanding behavior in firms that need to accommodate demands from retailers: *“[The supermarket chains] have specific reduction targets, and thus they expect us to have equally strict requirements or stricter […] since we work in the Scandinavian market, I see that the Norwegian chains have the least demands”—P3.*

Engagement with external stakeholders also goes beyond the value chain. We detected an active participation in (publicly funded) research projects focusing on sustainability aspects, revealing a dynamic capability to enable SOI through learning from other domains. While sourcing external knowledge, companies also participate in multi-stakeholder ecosystems and R&D partnerships [35, 98, 99], demonstrating an open innovation attitude. All the interviewed companies participate in one or several research projects in different strategic areas, such as food-waste reduction, renewable packaging, sustainable food choices, environmental footprint reduction, local food production, healthier and more sustainable diets, and automation, as shown by the following quote: *“We are very forward-thinking in terms of the sustainability perspective. We work a lot in research, where it is important for us with quite a few research projects, and we have really been that way all these years”—PP2.*

Many of the interviewed companies actively engage with policymakers to influence regulations and definitions that ideally support both sustainability and their core business. As one interviewee put it, “it is better to stay ahead of policy changes and try to be a part of shaping policy rather than waiting for the government to take action”—PP1. Viewing regulation as opportunity [10] describes an organizational transformation from pure compliance (adaptive firm behavior) to the willingness to make an impact (expanding firm behavior). Incumbent food companies linked to primary production (PP1, PP2, PP3) actively engage in policy forming process. In particular, this demonstrates how the highly regulated Norwegian agricultural sector [44] drives vertically integrated companies owned cooperatively by farmers to try to influence the political frame conditions that regulate their businesses. Although this aligns with Smink et al. [31] and Sovacool and Brisbois [62], who describe large incumbents influencing policies, we did not find any evidence of actions to create favorable lock-in situations but rather progressive thinking.

##### Embedding New Organizational Practices and Norms

Internal activities and processes to operationalize SOI were clearly mentioned as one of the most important and difficult processes requiring new dynamic capabilities. As mentioned in the “Strategic Shift in Focus from CSR to Sustainability Accountability” section, sustainability management has been increasingly integrated into existing organizations’ units between 2016 and 2020, sometimes as an evolution of a CSR role and sometimes as a completely new function. Indeed, many of the sustainability managers have been recruited specifically for this purpose. This is a clear example of expanding behavior, wherein sustainability is embedded as a strategic norm. External expectations were mentioned as a driver for this increased focus on SOI*:* *“The last one to two years was a turning point internally, so it is clear that, when the people out there change, it also changes how we work with [sustainability] internally”—PP1*. There is a constant effort to formalize roles and competencies both internally and through external networks, as also described by Klewitz and Hansen [13]. The need for new knowledge and skills is high, not only for sustainability but also for enabling technologies such as digitalization, automation, and artificial intelligence (AI). Expertise is drawn both from within the organization and collaboration with external stakeholders. External knowledge-sourcing can commonly come from suppliers: *“When we need expertise, we get it. From the supplier industry, from the ingredients industry, if we need it, we get what we need externally”—PP4.* Companies’ absorptive capacities and organizational learning facilitate the incorporation of sustainable values into their innovation strategy [35].

The integration of sustainability in the PD process was described by several companies and reflects a redesign of existing routines [13, 97]. Some incumbents introduced specific KPIs or a “traffic-light” system linked to sustainability early in the PD process (PP1, PP3, P2). The decision-making process for investments marks another example of changing internal processes. Interviewees mentioned that it is impossible to calculate return on investment (ROI) and depreciation time for sustainability investment and that SOI is often invisible to consumers and customers. This is different from traditional investments and requires different managerial decision processes, as one interviewee stated: *“We take a lot of costs to become more sustainable. Also, it is always a bit difficult to know what we get in return for it or to measure what we get in return. […] sometimes it's more about principle, because we think that those decisions or that cost we just have to carry, because we have to have sustainable raw materials, and we can have nothing else”—P2.*

Some respondents described the importance and challenges of focusing on the necessary cultural change when introducing a sustainability strategy: *“It takes a long time to get that mindset into a large organization with many autonomous units, so it requires some giga culture change”—PP3.* A top-down effort, good internal communication, and internal role models are crucial to achieve this cultural change. All interviewed companies are right in the middle of establishing new cultural norms fostering SOI, clearly recognizing the benefit of embedding SOI in every aspect of their operation: *“[B]y doing these things [sustainability initiatives], we become more efficient as a company … it can give us increased profitability … Yes, it makes us better as a company—it makes us a better employer in relation to more people wanting to work here, and we see that we get preferred in delivering our products to the market. It’s such a win–win situation. Working with sustainability is not a bad thing for either the economy or the company”—PP4.*

The need for organizational culture change towards SOI implies that sustainability becomes a new embedded strategic norm [14], requiring engagement of decision-makers and expanding dynamic capabilities that need to adjust over time [92]. This process is evolutionary and varies in pace depending on companies’ history and path-dependent logic, as Inigo and Albareda [35] also found.

#### Transforming Firm Behavior and System-Building

We identified several signs of transformative activities resembling SOI-integrated dynamic capabilities. The Norwegian food industry is increasingly adapting the terminology of “food systems.” Many of the interviewed companies are members of the Norwegian Technology Platform (NTP) “Food for Life,” a branch of the European Technology Platform (ETP).^5^ Following the ETP aim of achieving more sustainable food systems through research and innovation, the NTP and other networks in the Norwegian food industry (Fremtidsmat, Matfloken, Sustainability Hub, Packaging Association, FoodDrinkNorway) foster an active dialogue with the entire system to identify levers of change and form new collaborative platforms with diverse stakeholders [14]. Incumbents engage in open innovation activities to complement their competencies [71] even beyond their value chain. Strategic participation in research projects on radically new technologies and innovations signifies firms’ emerging ambidexterity [115]. Examples of the scope of such projects include cultured meat, hydrogen fuel, digitalization and automation, precision fermentation, AI, and genetic engineering.

The emergence of new circular business models exemplifies the reconfiguration and transformation of some of the incumbent firms [76]. New business paradigms aiming at achieving both circularity and CO_2_-neutral or -negative footprints have been rising and help firms be resilient towards disruptive innovations [28, 35]. PP4, for example, wants to multiply food production from the sea by utilizing species from lower in the food chain and utilize unexploited resources from fish farms, leading to safer and healthier food with reduced impact on the environment and climate. P2 established two new business units focusing on the utilization of seaweed and alternative proteins supporting a systemic sustainability transition. Already in 2000, PP2 spun off a company for by-product utilization and valorization of waste from animal production. As a new collaborative process platform, an industrial scale biorefinery for protein hydrolysates was established as a joint venture, and the companies participate in research projects. Circular usage of packaging materials is also high on the agenda for the entire Norwegian food industry and manifested in companies’ sustainability strategies. Such examples demonstrate a progressive change in the organization’s culture and a concrete tendency to build a system that spills over the extant value chain. Transforming firm behavior thus facilitates collaboration between the food industry and adjacent sectors and the adoption of radical innovation, redesigning current business models [27, 35].

## Conclusion and Implications

Our study confirms a high awareness and engagement in SOI among the incumbent firms in the Norwegian food sector, which play an important role as positive change agents in the sustainability transition [32, 67] and do not necessarily inhibit change as described by Béné [5], Markard et al. [56], and Geels [57]. There is no choice: incumbents must engage in SOI to avoid losing their shareholders, customers, and consumers.

Concerning the first research question, we found that definitions determine how a sector and individual company works with sustainability on a strategic level and operationalizes SOI. The definitions of sustainability and integration in firm strategy have evolved significantly in the past five years for all incumbent firms in this study and are strongly linked to the UN’s SDGs. There is an open dialogue and several initiatives in the Norwegian food system to both define and integrate sustainability [45, 54]. Materiality analysis centered on the individual company’s core business and position in the supply chain is a key tool to prioritize and communicate sustainability-oriented activities. This study contributes to clarifying the sustainability definitions for managers in the industry. Its insights could also offer a basis for policy development towards a more sustainable food system.

Regarding the second research question, this empirical study shows that adaptive and expanding SOI-integrated dynamic capabilities of sensing and seizing are well developed among the Norwegian incumbent food firms. Existing knowledge and capabilities are used to drive SOI, and new knowledge is sourced from external collaboration in research projects, open innovation activities, and strengthened supplier relationships. Some of the organizations are so large they exhibit all stages of SOI. The incumbent Norwegian food industry can be described as in a transitional state of operational transformation. Elements of operational optimization continue to be important for many players to keep their core business running at a cost-efficient level. Most innovations are incremental, not radically modifying business models, with some exceptions described in the previous section resembling transformative changes, such as new business models and engagement in research on radically new technologies. However, clear initiatives of moving away from a linear supply chain approach to a more systematic approach through food system collaborations have recently emerged. The consumer is an important part of the food system and driver for SOI. Publicly traded companies experience a strong demand for SOI and sustainability goals and reporting from their owners and shareholders. Cooperatively owned companies instead must consider the needs/lives of their owners (farmers) and consequently are somewhat limited in terms of radical transformations. We suggest that cumulative incremental changes will also lead to a sustainability transition, challenging the narrative of only radical or disruptive innovations leading to system change [58, 117].

Our research provides an integrated framework for SOI and dynamic capabilities in the context of the Norwegian food industry. We have applied the framework to a new empirical setting and broadened its dimensions by showing specific SOI-integrated dynamic capabilities and practices linked to food sustainability. We have illustrated the sustainability journey of the incumbents as a driving force for food system sustainability. In contrast to most studies that only include one to three companies, our study contains ten along the entire value chain, thus representing the mainstream Norwegian food industry. Moreover, the analytical framework constitutes a useful tool for understanding how firms develop SOI capabilities in other sectors or geographical contexts. Our results contribute to the current debate on the definition and operationalization of sustainability in the Norwegian food industry. We also offer a contribution for practitioners who would like to understand the relevance of different organizational practices in terms of dynamic capabilities to realize SOI. This may serve as a blueprint for managers and stakeholders to look at their current business models and internal strategies from a different angle and find potential new arrangements to enhance their environmental impact.

Limitations relate to the explorative nature of the study, which investigates one sector in one country representing a very peculiar case. Therefore, the results’ transferability should be viewed consciously and limited to a narrow context with comparable structural characteristics.

Given the urgency of the sustainability transition and the observed evolution in developing SOI and increasingly engaging in sustainable practices over time, it would be worthwhile to pursue further research in this domain. Future studies could explore radical innovation cases and how they influence the rest of the organization (i.e., ambidextrous organizations). Moreover, entrepreneurial research could investigate the drivers of the cultural change towards sustainability within well-established incumbent firms; young and/or start-up companies can also represent useful cases to detect further emerging innovative niches, and research should analyze their SOI activities. This research could extend to other countries and/or sectors, even with a quantitative follow-up study. Finally, it would be interesting to assess the impact of incumbents’ research project engagement for SOI-integrated dynamic capabilities development.

## Supplementary Information

Below is the link to the electronic supplementary material.

Supplementary file1 (DOCX 20 KB)

## Data Availability

The data presented in this study is available in the article (Table 1 and Figs. 1–3), as well as the direct quotations from interviews.

## Footnotes

1 The search was carried out on October 06, 2022.

2 From its realease, in 2016, the article from Adams et al. [14] has been cited 7814 times (excluding self-citations) in 442 articles. According to the citation report retrieved by Web of Science on October 6, 2022, the number of citations increased from 5 in 2016 to 2949 in 2021, reaching a peak. In 2022, the article has been already cited 2698 times, thus expecting to reach a value higher that 2021 at the end of the year.

3 For more information, please visit: https://www.helsedirektoratet.no/english/salt-and-the-salt-partnership.

4 For more information, please visit: https://www.helsenorge.no/kosthold-og-ernaring/kostrad/helsedirektoratets-kostrad (in Norwegian).

5 For more information, please visit: https://etp.fooddrinkeurope.eu/
