# Supplementary material for: Incumbents’ Capabilities for Sustainability-Oriented Innovation in the Norwegian Food Sector—an Integrated Framework
Source: Circ Econ Sustain. 2022 Nov 25:1–28. Online ahead of print. doi: 10.1007/s43615-022-00234-1 (PMC9702831; doi:10.1007/s43615-022-00234-1)
Supplement: Supplementary file 1 — Supplementary file1 (DOCX 20 KB) [file 43615_2022_234_MOESM1_ESM.docx]

**Interview guide SOI**

I have a guide, but please feel free to treat this as a conversation, we are always interested in stories and examples

Can you start with telling us about yourself, your background and your role in the organization?

**Sustainability**

- Tell us about sustainability in your organization
- How do you define sustainability?
- Do you have specific areas you focus on?
  - How do they correspond with relevant strategy?
- Are you working towards specific goals?
  - Which?
- Are you using any form of third-party control, certification, or environmental management tools?
- When did this work start? Can you try to date when sustainability entered strategic work?
- Who is responsible for sustainability in your organization?
  - To what extend is sustainability an integrated part of strategy work?
- How do you combine sustainability and value creation?
  - Is there a conflict?
- To what degree can you “impose demands” on other actors in your value chain? (for example, suppliers?)
- How do you break sustainability work down to the different areas (procurement, product development, production, innovation, marketing)?

**Innovation and product development**

- How is product development organized in your organization?
- What does your innovation strategy look like?
- In terms of product development – what role does sustainability play?
  - What is the ambition level?
- To what degree does motivation for sustainability come from consumer preferences?
  - How do you monitor consumer preferences?
- To what degree does motivation for sustainability come from policy or regulation?
  - How do you participate in policy work through hearings or industry associations?
- Can you give us any examples of product development resulting from sustainability goals? … or examples of strategic choices to include sustainability in innovation work?
- In your experience, is it easily possible to finance new projects or product development based on sustainability?
- Do you participate in research projects focused on sustainability?
- Can you give us an example of strategic choices made to include sustainability in innovation work?
- Do you have the right competencies to work with sustainability?
  - What new competencies might you need to be competitive with regards to sustainability in the future?
  - Which activities that will build new competencies do you participate in?
- To what degree is it possible to scrutinize / reconsider your core business?
  - What radical innovations can you think of within your company?
- Do you have any comments you would like to share?
